# An advanced understanding of the heterogeneous clinical features of “non-criteria” obstetric antiphospholipid syndrome: Two case reports and a literature review

**DOI:** 10.3389/fimmu.2023.1122127

**Published:** 2023-02-14

**Authors:** Xue Peng, Xi Tan, Aiyun Xing

**Affiliations:** ^1^ Department of Obstetrics and Gynecology, West China Second University Hospital, Sichuan University, Chengdu, China; ^2^ Key Laboratory of Birth Defects and Related Diseases of Women and Children (Sichuan University), Ministry of Education, Chengdu, China

**Keywords:** antiphospholipid antibodies, antiphospholipid syndrome, obstetric antiphospholipid syndrome, “non-criteria” obstetric antiphospholipid syndrome, seronegative antiphospholipid syndrome, pregnancy

## Abstract

Antiphospholipid syndrome (APS) is an acquired autoimmune disorder characterized by recurrent venous and/or arterial thrombosis and/or pregnancy complications, in the presence of elevated antiphospholipid (aPL) antibodies. APS in pregnant women is referred to as “obstetrical” APS (OAPS). The diagnosis of definite OAPS requires the presence of one or more typical clinical criteria and persistent aPL antibodies at least 12 weeks apart. However, the classification criteria for OAPS have generated wide discussion, with a growing impression that certain patients not fully meeting these criteria might be inappropriately excluded from the classification, which is known as “non-criteria” OAPS. We present here two unique cases of potentially lethal “non-criteria” OAPS, complicating severe preeclampsia, fetal growth restriction (FGR), liver rupture, preterm birth, refractory recurrent miscarriages, or even stillbirth. We further share our diagnostic search and analysis, treatment adjustment, and prognosis for this unusual antenatal event. We will also present a short review of an advanced understanding of the pathogenetic mechanisms of this disease, heterogeneous clinical features, and potential significance.

## Introduction

Antiphospholipid syndrome (APS) is a systemic autoimmune disorder with a wide range of vascular and obstetric manifestations associated with thrombotic and inflammatory mechanisms orchestrated by antiphospholipid (aPL) antibodies, namely, lupus anticoagulant (LA), anticardiolipin antibodies (aCL), or anti-β2 glycoprotein-I (aβ2GPI) antibodies (1). When thrombosis is the main clinical manifestation, it is called thrombotic APS (TAPS), and when pathological pregnancy is the main clinical feature, it is known as obstetric APS (OAPS) (1, 2). APS can occur alone, called primary APS; it can also coexist with other autoimmune diseases (most commonly systemic lupus erythematosus, SLE), defined as secondary APS (2). The clinical presentation spectrum of APS varies in severity and ranges from asymptomatic “carrier” for aPLs, seronegative APS, and OAPS to a life-threatening catastrophic APS (CAPS) (3).

OAPS is known to be associated with a significantly increased risk of pregnancy complications, including recurrent (three or more consecutive) pre-embryonic or embryonic miscarriages (<10 weeks), one or more otherwise unexplained fetal deaths (≥10 weeks of gestation), or delivery before 34 weeks for preeclampsia or placental insufficiency (4). Adequate medical management of OAPS can significantly improve pregnancy outcomes. However, there are many disputes in the diagnosis and treatment of OAPS, and insufficient understanding coexists with excessive diagnosis and treatment. Current guidelines state that the diagnosis of definite OAPS requires the presence of one or more typical clinical criteria and persistent aPL antibodies at least 12 weeks apart (**Table 1A**) (5). These diagnostic criteria of APS have been almost 20 years (developed in the late 1990s and revised in 2006) and have been little challenged (6). Concerns about the sensitivity and specificity of both the clinical and laboratory criteria are the subject of current debate among experts. With an advanced understanding of the pathogenetic mechanisms of this disease and heterogeneous clinical features, evidence is also accumulating on the potential clinical significance of more “non-criteria” clinical or laboratory manifestations of OAPS (7).

**Table 1A:** The international consensus (revised Sapporo) criteria for diagnosis of OAPS.

Clinical criteria	Laboratory criteria
1.Three or more sequential spontaneous abortions before 10th week of gestation, with maternal anatomic or hormonal abnormalities and paternal and maternal chromosomal causes excluded	1. LA present in plasma, confirmed on two or more occasions, at least 12 weeks apart
2.Unexplained fetal death of a morphologically normal fetus at or beyond the 10th week of gestation	2. aCL of Ig G and/or IgM isotype in serum or plasma, present in medium or high titer (i.e., > 40 GPL units or MPL units, or > the 99th centile), confirmed on two or more occasions, at least 12 weeks apart
3.Early birth before 34th week of gestation of a morphologically normal fetus due to eclampsia, severe pre-eclampsia or confirmed placental insufficiency	3.aβ2GPI of IgG and/or IgM isotype present in serum or plasma (in titer >the 99th centile), confirmed on two or more occasions at least 12 weeks apart

OAPS is diagnosed if at least one of the clinical criteria and one of the laboratory criteria are met.

OAPS, obstetric antiphospholipid syndrome; LA, lupus anticoagulants; aCL, anticardiolipin antibodies; aβ2GPI, anti-β2 glycoprotein-I antibodies.

**Table 1B:** The diagnostic criteria for NOAPS.

Non-criteria clinical manifestations	Non-criteria laboratory manifestations
1.Two sequential spontaneous abortions before 10th week of gestation, with maternal anatomic or hormonal abnormalities and paternal and maternal chromosomal causes excluded	1. Low positive aCL and/or aβ2GPI of IgG and/or IgM isotype present between the 95th and the 99th centile, confirmed on two or more occasions at least 12 weeks apart
2.Three non-sequential spontaneous abortions before 10th week of gestation, with maternal anatomic or hormonal abnormalities and paternal and maternal chromosomal causes excluded	2. Presence of intermittent aPLs (the interval is <12 weeks)
3.late preeclampsia, late premature birth, placenta abruption, placenta hematoma	
4.Two or more unexplained *in vitro* fertilization failures	

NOAPS is diagnosed if the patient has a) one of non-criteria clinical manifestations and one of international consensus laboratory criteria or b) one of international consensus clinical criteria and one of non-criteria laboratory manifestation.

NOAPS, non-criteria obstetric antiphospholipid syndrome; LA, lupus anticoagulants; aCL, anticardiolipin antibodies; aβ2GPI, anti-β2 glycoprotein-I antibodies.

In the present study, we aim to report two rare and potentially lethal cases of “non-criteria” OAPS (NOAPS), describing their clinical features, treatment, and outcomes.

## Case report

Case 1 was a 36-year-old woman, 28 + ^1^ weeks of pregnancy, gravida 2, para 0, with a previous history of induced abortion. She was admitted to the emergency department of our hospital due to a sudden onset of significantly increased blood pressure. The patient’s past history was unremarkable. The patient had regular prenatal care, and everything seemed to be normal at first. However, she developed edema of both lower limbs 1 week ago and was recently found to have a blood pressure of 140–150/90–100 mmHg. She reported no dizziness, headache, or visual disturbance. In the emergency room, the patient had proteinuria 3+ and blood pressure of 170/94 mmHg, so the preliminary diagnosis of “severe preeclampsia” and emergency admission were made.

**Table 2** resumes the patient’s laboratory evolution during hospitalization. Serial obstetric ultrasound scanning showed that the fetal growth was significantly slowed in the corresponding gestational weeks, and fetal growth restriction (FGR) was considered. Immunoassay further indicated the formation of excessive immune antibodies associated with APS and undetermined Sjogren’s syndrome (SS). Although the patient had no history of pregnancy morbidity, severe preeclampsia and FGR had been diagnosed in this pregnancy. Combined with laboratory examination, NOAPS secondary to some autoimmune disorders was then considered in the following specialist consultation.

**Table 2:** Case 1 laboratory evolution during hospitalization.

	28+1 week	30+6 week	31 week 10:00	31 week 12:00	31 week 14:00	31 week 14:04 surgery	2 h after operation	3 days after operation	Reference range
WBC	5.5	6.9	12	12.2	12.5	/	18.3	14.5	4–10×10^9^/L
HB	119	140	112	100	68	/	113	92	110–150 g/L
PLT	147	208	217	213	148	/	89	116	100–450×10^9^/L
ALT	51	208	160	/	/	/	132	220	<49 U/L
AST	77	89	70	/	/	/	92	108	<40 U/L
TB	10.4	7.8	9.2	/	/	/	12.6	15.6	5–23 μmol/L
DB	4.5	3.6	4.3	/	/	/	7.2	9.6	<8 μmol/L
ID	4.1	4.2	4.9	/	/	/	5.4	6	<17 μmol/L
ALB	30.5	28.5	25.1	/	/	/	23.5	27.4	34–55 g/L
LDH	261	257	206	/	/	/	422	312	120–246 U/L
UREA	4.21	9.19	11.19	/	/	/	10.37	9.13	2.6–7.5 mmol/L
Cr	68	80	101	/	/	/	96	78	30.4-73 μmol/L
Proteinuria	6.058	8.873	/	/	/	/	/	0.767	<0.14 g/24 h
PT	12.1	11.5	/	12.1	13.3	/	12.3	12.4	7.6–13.6 s
APTT	32.1	30.8	/	28.1	42.6	/	32	22.5	16.9–36.9 s
FIB	621	526	/	413	253	/	314	651	200–400 mg/dl
DDI	0.58	0.27	/	–	–	/	–	9.27	<0.55 mg/L
ATIII	98	107	/	–	–	/	–	89	75%–125%
LA	+	+	/	–	–	/	–	+	Negative
aCL IgG/IgM	–	–	/	/	/	/	/	–	<20 RU/ml
aβ2GPI IgM	33.6	27.84	/	/	/	/	/	8.08	<20 RU/ml
aβ2GPI IgG	243.18	155.95	/	/	/	/	/	80.12	<20 RU/ml
ANA	1:1000	1:320	/	/	/	/	/	1:100	Negative
anti-SSA(Ro)	300	275.9	/	/	/	/	/	190.1	<20 RU/ml
anti-Ro52	303	186.9	/	/	/	/	/	92.9	<20 RU/ml
anti-NU	62.2	33.6	/	/	/	/	/	20.4	<20 RU/ml
anti-dsDNA	–	–	/	/	/	/	/	–	Negative
anti-Smith	–	–	/	/	/	/	/	–	<20 RU/ml
C3	1.26	1.3	/	/	/	/	/	1.28	0.9–1.8g/L
C4	0.3	0.2	/	/	/	/	/	0.3	0.1–0.4g/L
CD4+/CD8+	0.6	0.7	/	/	/	/	/	0.7	1.4–2.0

WBC, white blood cell; HB, hemoglobin; PLT, platelet count; ALT, alanine aminotransferase; AST, aspartate transaminase; TB, total bilirubin; DB, direct bilirubin; ID, indirect bilirubin; ALB, albumin; LDH, lactate dehydrogenase; Cr, creatinine; PT, prothrombin time; APTT, activated partial thromboplastin time; FIB, fibrinogen; DDI, D-Dimer; ATIII, antithrombin III; LA, lupus anticoagulants; aCL, Anticardiolipin antibodies; aβ2GPI, Antiβ2glycoprotein-I antibodies; ANA, antinuclear antibodies; anti-NU, anti-nucleosome antibodies.

The prescription was to give prednisone 20 mg qd orally, tacrolimus 0.5 mg qd orally, and hydroxychloroquine (HCQ) 200 mg qd orally. In addition, based on oral administration of 100 mg low-dose aspirin (LDA) once a day, 4,000 IU low molecular weight heparin (LMWH) was injected subcutaneously at q12h for treatment.

At 31 weeks of pregnancy, the patient complained of sudden and continuous abdominal pain accompanied by a decrease in blood pressure and hemoglobin level. Emergency ultrasound showed a large amount of peritoneal effusion. Considering the risk of intraperitoneal hemorrhage, an emergency laparotomy was immediately performed.

**Figure 1:**
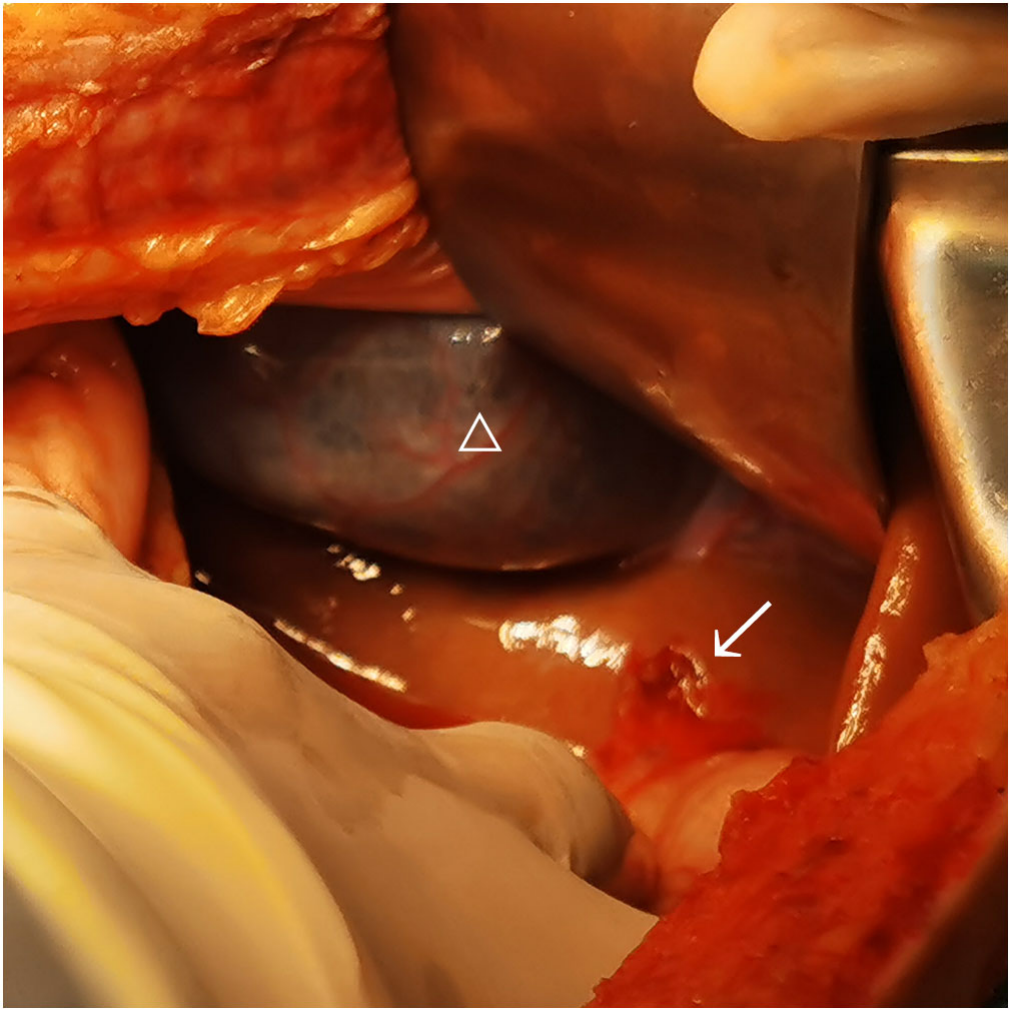
Hepatic rupture of case 1. “△” represents gallbladder; “→” represents hepatic rupture.

During the operation, a large amount of blood accumulation (approximately 2,800 ml) and blood clots were seen in the abdominal cavity, but there were no signs of uterine rupture or placental abruption. A live female baby weighing 1,030 g was delivered by emergency cesarean section. Apgar scores were 3–6–8 points at 1, 5, and 10 min, respectively. The baby was then transferred to the neonatal intensive care unit (NICU) immediately. The surgeon carefully explored and found a rupture of 1.5 cm accompanied by active bleeding on the liver surface just below the bottom of the gallbladder (**Figure 1**). After surgery, the patient was sent to the ICU ward for further treatment. The patient recovered well and was discharged 8 days later, and the newborn recovered and was discharged 3 weeks later. The patient was then advised to continue to use LMWH (4,000 IU qd) for 6 weeks after delivery to reduce the risk of maternal thrombosis. The placental histological examination showed mild chorioamnionitis, with the presence of hypermature avascular choral villi with multiple infarctions, vacuolization, and inter-/intra-fibrin deposition and focal calcifications.

Three months after delivery, the patient was re-evaluated at the Internal Medicine and referred to an immunologist. Investigations showed positive Schirmer test and persistently elevated aPLs, ANA (homogeneous pattern), anti-SSA(Ro), and anti-Ro52 antibodies. The patient was then diagnosed with OAPS secondary to SS.

Case 2 was a 31-year-old woman, 30 + ^4^ weeks of pregnancy, gravida 6, para 1, with four consecutive miscarriages (**Table 3**). The patient was found to have a high titer of anti-phosphatidylethanolamine (aPE) antibody in the past. Laboratory workup was repeated at least 12 weeks apart and continued to be negative for criteria aPLs, and screening for thrombophilia (e.g., protein C/S deficiency and antithrombin III deficiency) and other autoimmune diseases (e.g., SLE and SS). Besides, the causes of conceptus aneuploidy, infectious diseases, uterine malformations, parental karyotype abnormalities, and maternal endocrinopathies had been ruled out.

**Table 3:** Pregnancy history of case 2.

Gravida	Years	Pregnancy outcome	Immunoassay	Therapeutic regimen	Conceptus aneuploidy
1	2016	at the 6th week of gestation biochemical pregnancy	/	None	None
2	06/2017	at the 10th week of gestation fetal loss	/	progesterone	None
3	09/2017	at the 7th week of gestation spontaneous abortion	/	progesterone	None
4	2019	at the 9th week of gestation spontaneous abortion	aPE (+)	progesterone, traditional Chinese medicine	Negative
5	2020	at the 29th week of gestation FGR, AEDV/REDV, stillbirth	aPE (+)	HCQ (200 mg/day), LDA (75 mg/day), LMWH (4,000 IU/day)	Negative
6	2022	at the 34th week of gestation FGR, AEDV, emergency CS, neonate was healthy	aPE (+)	HCQ (400 mg/day), LDA (100 mg/day), LMWH (6,000 IU/q12h), prednisone (5 mg/day), IVIg (400 mg/kg/month)	Negative

FGR, fetal growth restriction; AEDV, absence of end-diastolic flow velocities; REDV, reversal of end-diastolic blood flow velocity; CS, cesarean section; aPE, anti-phosphatidylethanolamine antibody; ANA, antinuclear antibodies; aPLs, antiphospholipid antibodies; HCQ, hydroxychloroquine; LMWH, low molecular weight heparin; IVIg, intravenous immunoglobulin; LDA, low-dose aspirin.

With concerns about unexplained recurrent miscarriages and aPE positivity, NOAPS was suspected before the fifth pregnancy of the patient (**Table 3**). Accordingly, at the confirmation of early pregnancy (singleton pregnancy with natural conception), HCQ treatment was initiated and maintained (at the dose of 200 mg/day), and prophylactic treatment with LDA (75 mg/day) and LMWH (4,000 IU/day) was added, too. Despite this, color Doppler investigation of the umbilical artery flow indicated an absence/reversal of the end-diastolic flow velocities (AEDV/REDV) at 21 weeks of pregnancy. With rising fetoplacental circulatory impedance, fetal growth was significantly delayed, too. The fetal condition deteriorated sharply, and the patient refused to terminate the pregnancy until the fetus died *in utero* at the 29th week of pregnancy. The dead fetus weighing only 905 g had no obvious deformity. Fetal gene chip analysis was also normal. No histological evaluation of the fetus and placenta was available.

**Table 4** resumes the patient’s laboratory findings during this pregnancy. Before this pregnancy, laboratory workup was repeated at least 12 weeks apart, which continued to be negative for criteria aPLs, but “non-criteria” aPL antibody (aPE) was persistently positive. Considering the diagnosis of refractory and seronegative OAPS in this case, based on HCQ treatment for a long-term basis, at the confirmation of pregnancy (singleton pregnancy with natural conception), HCQ treatment was maintained (at the dose of 400 mg/day), and she underwent an add-on treatment with LDA (100 mg/day), LMWH (6000 IU/q12h), and prednisone (5 mg/day). In addition, starting from the sixth week of gestation, prophylactic IVIg co-therapy was started at a dose of 400 mg/kg/month (15 g for two consecutive days monthly).

**Table 4:** Case 2 laboratory findings during this pregnancy.

	Before pregnancy	12+3 week	30 week	33+6 week	42 days after delivery	Reference range
WBC	7.8	10.1	9.8	11.1	6.3	4–10×10^9^/L
HB	132	122	103	115	129	110–150 g/L
PLT	233	198	173	152	205	100–450×10^9^/L
ALT	33	58	49	39	32	<49 U/L
AST	29	44	41	38	22	<40 U/L
TB	7.1	6.3	7.9	8.5	9.6	5–23 μmol/L
DB	3.1	6.4	5.5	4.2	3.7	<8 μmol/L
ID	5.2	3.6	4.1	4.6	6.1	<17 μmol/L
ALB	50.1	48.3	35.7	36.2	52.2	34–55 g/L
LDH	144	189	208	213	158	120–246 U/L
UREA	3.21	3.88	4.02	3.19	4.07	2.6–7.5 mmol/L
Cr	54	34	45	47	63	30.4–73 μmol/L
Proteinuria	0.08	0.12	0.1	0.11	0.06	<0.14 g/24 h
PT	11	10.5	10.7	11.2	11.5	7.6–13.6s
APTT	31.1	28.1	27.3	27.4	32.3	16.9–36.9s
FIB	321	526	573	499	342	200–400 mg/dl
DDI	0.14	0.51	2.33	3.05	0.25	<0.55 mg/L
ATIII	112	98	101	89	106	75–125%
PC	110	88	91	85	109	70–140%
PS	94	85	73	74	100	76–135%
TSH	2.494	2.151	1.94	/	/	0.55–4.78 mIU/L
TORCH	–	–	–	/	/	Negative
LA	–	–	–	–	–	Negative
aCL IgG/IgM	–	–	–	–	–	<20 RU/ml
aβ2GPI IgM	–	–	–	–	–	<20 RU/ml
aβ2GPI IgG	–	–	–	–	–	<20 RU/ml
aPE	34.12	25.81	31.2	29.65	35.28	0–15 U/ml
ANA	–	–	–	–	–	Negative
anti-SSA(Ro)	–	–	–	–	–	<20 RU/ml
anti-Ro52	–	–	–	–	–	<20 RU/ml
anti-NU	–	–	–	–	–	<20 RU/ml
anti-dsDNA	–	–	–	–	–	Negative
anti-Smith	–	–	–	–	–	<20 RU/ml
C3	1.31	1.29	1.25	1.19	1.32	0.9–1.8g/L
C4	0.18	0.27	0.31	0.29	0.16	0.1–0.4g/L
CD4+/CD8+	1.6	1.5	0.8	0.6	1.7	1.4–2.0

WBC, white blood cell; HB, hemoglobin; PLT, platelet count; ALT, alanine aminotransferase; AST, aspartate transaminase; TB, total bilirubin; DB, direct bilirubin; ID, indirect bilirubin; ALB, albumin; LDH, lactate dehydrogenase; Cr, creatinine; PT, prothrombin time; APTT, activated partial thromboplastin time; FIB, fibrinogen; DDI, D-Dimer; ATIII, antithrombin III; PC, protein C; PS, protein S; TSH, thyroid-stimulating hormone; TORCH, Toxoplasma, Others, rubella virus, cytomegalovirus, herpes virus; LA, lupus anticoagulants; aCL, anticardiolipin antibodies; aβ2GPI, anti-β2 glycoprotein-I antibodies; aPE, anti-phosphatidylethanolamine antibodies; ANA, antinuclear antibodies; anti-NU, anti-nucleosome antibodies.

At 30 weeks of gestation, the color Doppler ultrasound showed that the fetal growth index was less than the 10th percentile of the corresponding gestational age and intermittent AEDV. No abnormality was found in the chromosome microarray analysis of the fetus.

At 34 weeks of gestation, due to the non-reactivity of fetal cardiotocography and persistent AEDV, an emergency cesarean section was performed, and a live female baby weighing 1,900 g was delivered, with an Apgar score of 8–9–10 at 1, 5, and 10 min, respectively. The baby was then transferred to the NICU. The mother was discharged on the third day after the operation. LMWH (4000 IU qd) was continued for 1 week after delivery to reduce the risk of maternal thrombosis. Placental examination showed immature singleton placenta, mild villitis, and villous interstitial inflammation with multiple infarctions.

At 6 months of postpartum evaluation, no complications were seen in the mother or child. During the follow-up (8 months), the patient did not present any signs or symptoms of active systemic disease, and the child was healthy.

## Discussion

The two cases reported here were both rare but potentially lethal “non-criteria” OAPS, leading to severe preeclampsia, FGR, preterm birth, hepatic rupture, recurrent miscarriages, or even stillbirth. Definite OAPS, fulfilling at least one clinical and one laboratory criteria of the updated Sapporo classification criteria (**Table 1A**), can occur in association with other autoimmune diseases (secondary OAPS) or in its primary form (primary OAPS) (5, 8). However, the classification criteria for OAPS have generated wide discussion, with a growing impression that certain patients not fully meeting these criteria might be inappropriately excluded from the classification (9). Nonetheless, these “non-criteria” patients are heterogeneously defined across the literature (4).

An accepted diagnosis of “non-criteria” OAPS is considered to be present if the patient has (7) a) a combination of non-criteria clinical manifestations with international consensus laboratory criteria or b) international consensus clinical criteria with a non-criteria laboratory manifestation (**Table 1B**). Non-criteria clinical manifestations include two unexplained miscarriages, three non-consecutive miscarriages, late preeclampsia, placental abruption, late premature birth, or two or more unexplained *in vitro* fertilization failures. Non-criteria laboratory manifestations are proposed as low positive aCL and aβ2GPI antibodies (95th–99th centile) and the presence of intermittent aPLs (<12 weeks apart). However, the two reported cases do not adequately fulfill the diagnostic criteria mentioned above, indicating potential types/subsets of “non-criteria” OAPS (1, 9).

Preeclampsia complicates 2%–8% of all pregnancies with high maternal and perinatal morbidity and mortality (10). It is difficult to identify pregnant women at high risk of preeclampsia before it presents clinically, such as in case 1. However, patients with early-onset preeclampsia or FGR should be screened for potential risk factors carefully, such as chronic hypertension, diabetes, SLE, APS, or chronic kidney disease (11–13). Most experts believe that early delivery for severe preeclampsia and/or placental insufficiency are more specific clinical features of OAPS (6). The mechanisms through which APS contributes to preeclampsia may be explained in part by the interaction between aPLs and endothelial cells, leading to thrombosis and microangiopathy (13). aPLs are also potential inducers of the peri-implantation inflammatory environment that can lead to an abnormal invasion of the trophoblast in the decidua and spiral arteries (4). This hypercoagulable state and placental spiral artery vasculopathy are associated with placental infarction, fetal growth restriction, fetal loss, and preeclampsia (10). A recent prospective, case–control study of women delivered for severe preeclampsia or placental insufficiency found that over 10% of cases were positive for aPLs compared with <2% of controls (14). Case 1 was characterized by early-onset preeclampsia and FGR, and laboratory examination suggested a high-risk aPLs profile and the presence of high titer autoantibodies associated with other autoimmune disorders. Identifying the presence of factors associated with high risk for thrombotic and obstetric events is critical in patient management. A major risk factor is the high-risk aPLs profile, including any of the following (1): the presence of LA, or of double (any combination of LA, aCL antibodies or aβ2GPI antibodies) or triple (all three subtypes) aPLs positivity, or the presence of persistently high aPLs titers. Additional risk factors for clinical events are the coexistence of other systemic autoimmune diseases, especially SLE or SS, and a history of thrombosis and/or OAPS. In addition, the laboratory findings of case 1 might suggest the diagnosis of Sjogren’s syndrome rather than SLE. The differential diagnosis of SS was based on the evidence of immunoassay and clinical manifestations: anti-SSA positive, anti-dsDNA and anti-Smith negative, and normal C3 or C4 level. In addition to antibodies, the patient had no symptoms of multi-system damage. Because the patient had no previous history of adverse pregnancy and could not wait for 12 weeks to review the antibody spectrum, she did not meet the diagnostic criteria of definite OAPS or NOAPS (**Tables 1A, B**) for the time being. However, given the high maternal–fetal morbidity or mortality caused by severe preeclampsia, FGR, and high-risk aPLs profile complicated with other autoimmune disorders, medical interventions had been promptly considered. During the 3 months follow-up after delivery, laboratory workup indicated persistent positivity of aPLs profile and autoantibodies. The patient was finally diagnosed with definite OAPS secondary to SS.

Common autoimmune connective tissue diseases (CTDs) closely related to pregnancy morbidity include SLE, APS, and SS. The incidence of CTDs is high in women of childbearing age, and some patients have been diagnosed with CTDs several years before pregnancy. Most patients are treated with glucocorticoids or immunosuppressants for a long time (15, 16). Pregnancy can easily lead to the worsening of CTDs. Clinicians should be aware that CTDs, especially SLE and APS, can also put pregnancies at significantly high risk of placental insufficiency, such as FGR and preeclampsia (17, 18). The best pregnancy outcome can be achieved by performing multidisciplinary management and paying attention to the evaluation and treatment of basic diseases. The use of LDA as preeclampsia prevention in CTDs pregnancy may be the best choice now. For APS patients with SLE or other autoimmune diseases, on the basis of immunotherapy, treatment with LDA and the preventive or therapeutic dose of LMWH should be maintained throughout pregnancy according to the patient’s risk.

The pathological mechanism of hypertensive disorder complicating pregnancy is systemic vasospasm. Periportal hemorrhage and intravascular fibrin deposition play an important role in hepatic sinus obstruction and massive intravascular congestion, leading to elevated intrahepatic pressure and infarction development, and subcapsular hematoma and intraparenchymal hemorrhage (16). The hypercoagulable state and microthrombosis related to OAPS may further aggravate this situation (17). In addition, the application of high-dose LMWH will increase the risk of spontaneous visceral hemorrhage, although it is extremely rare (18). Therefore, the use of LMWH during pregnancy needs to strictly follow medical indications in order to benefit the mother and the fetus. At the same time, the side effects of heparin such as bleeding should be closely monitored in clinical use.

Given the clinical manifestations of case 2, which are four consecutive miscarriages and a stillbirth at the 29th week of gestation, the patient undoubtedly fulfilled the international consensus clinical criteria for the definite OAPS. However, the absence of “criteria” aPLs prevented this diagnosis, even though this case hardly fit any other known coagulopathy, thrombophilias, or disorders. In addition, for patients with recurrent abortion or fetal loss, infectious diseases, maternal anatomy or hormone abnormalities, and embryonic, paternal, and maternal chromosome causes should be excluded first in the differential diagnosis.

Clinically, there are women fulfilling only the clinical criteria related to OAPS, but no laboratory criteria are present. It poses a diagnostic challenge for physicians and supposes extra stress for patients. These cases have negative or low titers of aPLs, or only once or intermittent positive for aPLs, or showing positivity for “non-criteria” aPLs, which are known as seronegative APS or subtypes of “non-criteria” APS (4). Seronegative APS (SN-APS) was initially defined in a 2003 publication by Hughes and Kamashta (19). Rodriguez-Garcia (20) and Gilberto (9) later described SN-APS as patients with clinical manifestations fulfilling APS classification criteria, plus the presence of “non-criteria” manifestations (at least one obstetric or one major non-obstetric or two minor non-obstetric), with persistently negative aPLs (at least two determinations 12 weeks apart), and exclusion of other thrombophilias that justify the whole clinical presentation. However, the existence or diagnostic criteria of SN-APS is still under debate (21).

Accordingly, case 2 could be an SN-APS. In these cases, the laboratory results for “criteria” aPLs were consistently negative, and it was assumed that they were false negatives due to “consumption” or the presence of other aPL antibodies not usually tested (22). In clinical practice, there are often discrepancies between antibody levels and clinical expression. Routine screening tests for “criteria” aPLs may miss some cases because of the presence of “non-criteria” aPLs, and it is also possible that previously positive aPLs become negative or low titers, either acutely by “consumption” during an acute thrombotic episode, or slowly, like nephrotic syndrome over time (19, 22). Other scenarios should also be considered to explain this issue (4). First is the aPLs variability due to treatment administration, e.g., heparin, prednisone, or HCQ use. Second is pregnancy and aPLs variability. Pregnancy may induce a fluctuation, reduction, or fall in aPLs titers. This situation may be more frequent in cases treated with low-dose prednisone, HCQ, or heparin. Thus, a negative aPLs test during pregnancy, particularly if heparin is administered, should not rule out the diagnosis of OAPS. Third are laboratory or technical limitations in ELISA tests. In fact, the disparity for aPLs tested by ELISA, either commercial kits or “homemade” methods that lead to high inter-laboratory, intra-laboratory, or inter-method variability, has been recognized.

In view of the above, the clinical team considered the diagnosis of case 2 as refractory and seronegative OAPS, and the patient was treated accordingly, with good maternal and infant results (23). During pregnancy, the prescription was adjusted to give therapeutic doses of LMWH, IVIg co-therapy, low-dose prednisone, LDA, and HCQ maintenance. The occurrence of AEDV was successfully delayed by nearly 9 weeks.

The management of women with OAPS includes close surveillance and tailored treatment based on risk stratification before, during, and after pregnancy to optimize the maternal and fetal outcomes at maximum. A well-balanced team that includes obstetricians, rheumatologists, or immunologists is mandatory (4). Currently, the gold standard of definite OAPS treatment is LDA combined with LMWH at prophylactic or full doses individually from the moment of the positive pregnancy test. In patients with previous thrombotic events, full unfractionated or LMWH doses or anti-vitamin K therapy should be administered (4). In severe cases, the administration of prednisone, immunosuppressive drugs, and/or intravenous immunoglobulins have been used with the intention to suppress aPLs (4, 10). In addition, maternal morbidity and mortality depend on the severity of OAPS, whereas perinatal prognosis depends primarily on gestational age.

In conclusion, clinical practice in OAPS is highly variable, in part because it is a rare disorder, and the understanding of its diagnosis, clinical manifestations, and management are continuously advancing. There is great heterogeneity among studies on the criteria used to define OAPS/NOAPS and the treatment used over the past decades (1). These factors make it often difficult to apply the “best” interventions. Moreover, high-quality studies related to OAPS will necessarily require “rare disease” approaches (6). The two rare cases reported here further demonstrate that NOAPS can result in severe maternal or neonatal morbidity and mortality. Therefore, we emphasize that the diagnosis of NOAPS should be based on the clinical manifestations of adverse pregnancy. We recommend that women with early-onset severe preeclampsia and/or a history of recurrent pregnancy loss should be carefully evaluated for OAPS or its subsets, and closely monitored throughout pregnancy.

## Data availability statement

The original contributions presented in the study are included in the article/supplementary material. Further inquiries can be directed to the corresponding author.

## Ethics statement

Written informed consent was obtained from the individual(s) for the publication of any potentially identifiable images or data included in this article.

## Author contributions

XP, XT, and AX: study concept and design the overall study. XP and XT: analyzed and interpreted the patient data. XP and AX: drafted the manuscript. All authors contributed to the article and approved the submitted version.
